# A survey on postgraduate pharmacology education in India

**DOI:** 10.4103/0253-7613.68439

**Published:** 2010-08

**Authors:** Ananya Chakraborty

**Affiliations:** Department of Pharmacology, Vydehi Institute of Medical Sciences, Whitefield, Bangalore - 560 066, India. E-mail: dr_ananya@yahoo.com

Sir,

Recent advances have opened up exciting career opportunities for pharmacologists. However, there is a belief that the current postgraduate pharmacology curriculum has not kept pace with the advances.[1,2] A survey was conducted to assess the opinion of pharmacologists toward postgraduate pharmacology education in India. The questionnaire was forwarded to the online communities (indpharm and genxpharm), and distributed during the IPS conference, 2009. A total of 113 participants (Online: 47; IPS Conference: 66) completed the survey. The responses are discussed below.

**Figure 1:**
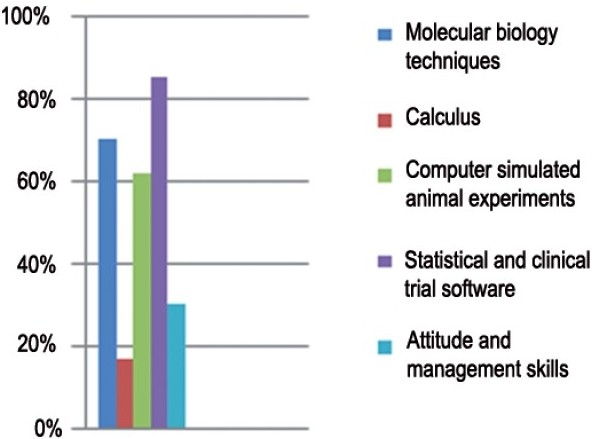
Additional training required for employability of pharmacology PG students

*Curriculum focus*: It was felt by 96% respondents that pharmacology curriculum lays emphasis only on becoming better undergraduate teachers. Eighty three percent respondents felt that the curriculum provides inadequate practical training on research methodology. Seventy four percent respondents mentioned that a primary challenge is to train the students in the recent advances. The participants suggested additional trainings required for employability that are shown in Figure 1.

*Institutions*: Most institutes are not equipped with required infrastructure as opined by 93% respondents. Eighty seven percent felt that the faculty members are not trained in recent advances. A collaborative and positive research atmosphere was lacking as opined by 55% of respondents, while 65% opined that their institutions do not provide funds for registration or travel allowances to attend or present their scientific research at national conferences.

*Career options*: The participants were aware of their role as teachers (98%) and as a functionary in pharmaceutical (72%) and medical writing industry (52%). Only 13% knew about the opportunities in BA, BE studies, and pharmacovigilance.

*Research preference*: Participants showed interest in preclinical (9%) and clinical research (34%) alone, while other participants mentioned interest in both preclinical and clinical research.

*Training programs*: The participants felt that interactive workshops should be compulsory for postgraduates. There should be assignments, practical exposure, and assessments. Sixty two percent mentioned that departmental seminars and journal clubs should have assessments and periodic reviews of the PG students.

*Examination pattern*: This is theory oriented with less of practical approach as felt by 91% respondents. Also, the PG dissertation topics are repetitions of previous studies leading to few noteworthy scientific contributions.

*Need of the hour*: The curriculum should be modified to accommodate recent advances. There should be internship opportunities, industry-academia, and inter-academia collaborations. Recent advances should be included in the vast curriculum by means of a credit-based modular approach where each training program or exposure-stint could earn the student some credit points. The accumulation of credit points could be a pre-requisite for taking the final examination. Also, the evaluation process for the post-graduates requires a relook. The written examination could include therapeutic case scenarios. Keeping in mind the emerging role of pharmacologists, the practical examination could evaluate additional skills such as screening of clinical trial subjects, informed consent, role play as an ethical committee member etc. Also, at least one examiner could be chosen from a national panel.

To conclude, learning requires academic freedom, good infrastructure, and a collaborative and positive research atmosphere, ‘Where the mind is without fear and the head is held high…’. We need to ensure that.
